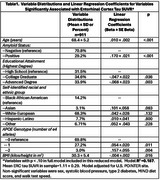# Cognitive Reserve is Associated with Entorhinal Cortex Tau Burden in an Older, Cognitively Unimpaired Clinical Trial Cohort (U.S. POINTER)

**DOI:** 10.1002/alz70857_104493

**Published:** 2025-12-25

**Authors:** Valory Pavlik, Christopher J. Weber, Joseph C. Masdeu, Laura D Baker, Melissa Yu, Michelle York, Rachel A. Whitmer, Susan M. Landau, Theresa M. Harrison, Thomas Monroe Holland, Laura Lovato

**Affiliations:** ^1^ Baylor College of Medicine, Houston, TX, USA; ^2^ Alzheimer's Association, Chicago, IL, USA; ^3^ Houston Methodist Research Institute, Houston, TX, USA; ^4^ Wake Forest University School of Medicine, Winston‐Salem, NC, USA; ^5^ University of California, Davis, Davis, CA, USA; ^6^ Neuroscience Department, University of California, Berkeley, Berkeley, CA, USA; ^7^ University of California, Berkeley, Berkeley, CA, USA; ^8^ Rush Institute for Healthy Aging, Chicago, IL, USA

## Abstract

**Background:**

High cognitive reserve (CR) is associated with a reduced dementia risk. and better cognitive performance for given levels of Alzheimer's disease (AD) pathology. We hypothesized that CR is associated with baseline amyloid and tau pathology in the U.S. Study to Protect Brain Health through Lifestyle Intervention to Reduce Risk (U.S.POINTER) Trial cohort.

**Method:**

Participants were 60 to 79 years old, cognitively unimpaired, sedentary, did not follow a Mediterranean‐DASH (MIND) diet, and had two or more other dementia risk factors. Of 2011 randomized, 52% consented to an ancillary imaging study that included amyloid and tau positron emission tomography (PET). Odds ratios (OR) and beta coefficients from logistic regression and linear regression models respectively were calculated to test whether higher EDU, an accepted CR measure, predicted lower risk of amyloid positivity, and lower entorhinal cortex (ERC) and meta‐temporal region of interest (meta‐ROI) tau standardized uptake value ratio (SUVR). Models were adjusted for age, sex, race/ethnicity, family history of dementia, APOE genotype, metabolic, dietary and physical fitness measures, and U.S.POINTER site.

**Result:**

911 U.S.POINTER imaging study participants had complete imaging data. Mean age was 68.4 ±5.24 years, 61.3% were female, 31.5% completed only high school (HS), 34.6% were college graduates (CG), 33.9% had advanced degrees (AdD) and 29.2% were amyloid positive on PET. Predictors of amyloid positivity included age (OR=1.09_1‐year increment_, 95% CI = .06,1.13, *p* < .001), race‐ethnicity (1.67_white vs black/AA_, 95% CI=0.99‐2.82, *p* = .054; 2.31_other vs black/AA,_ 95% CI=1.10, 4.84, *p* = .024), number of APOE e4 alleles (OR_1 copy_=3.01, 95% CI=2.16, 4.19, *p* < .001; OR_2 copies_=10.66, 95% CI = 4.40, 25.82, *p* < .001), and memory loss in mother (OR=1.47, 95% CI=1.06,2.04, *p* = .020). Higher CR was significantly associated with lower ERC tau SUVR (Beta _CG vs HS_ = ‐.047±.02, *p* = .038; Beta _AdD vs HS_ = ‐.068±.023, *p* = .003). EDU was unrelated to amyloid status or meta‐ROI tau.

**Conclusion:**

In the U.S.POINTER Cohort, higher CR predicted lower tau burden in the ERC, a brain area first affected by tau deposition. Meta‐ROI tau may be too low in this cognitively unimpaired sample to reveal associations. The findings suggest a possible role of CR in conferring biological resistance to AD pathology.